# Evaluation of various internal standards for quantification of dextromethorphan and diphenhydramine in plasma: a fatal overdose case of a mid-teenager caused by personally imported and over-the-counter medicines

**DOI:** 10.1007/s11419-025-00736-1

**Published:** 2025-08-01

**Authors:** Yujin Natori, Hayato Miura, Takashi Yoshimoto, Akira Ishii

**Affiliations:** 1 Department of Forensic Medicine and Bioethics, Nagoya University Graduate School of Medicine, 65 Tshrumai-cho, Showa-ku, Nagoya, Japan; 2 Nagoya University Hospital, 65 Tshrumai-cho, Showa-ku, Nagoya, Japan

**Keywords:** Diphenhydramine, Dextromethorphan, Overdose, Internal standards, Liquid chromatography-tandem mass spectrometry, LC/MS/MS

## Abstract

**Purpose:**

Over-the-counter medicines are commonly used for recreational and suicidal overdoses, a global problem. Some of these are easily obtained via the Internet. In cases of intoxication, drug quantification is necessary to estimate the cause of death. Stable isotope compounds are recommended as internal standards (IS) for analyzing drugs; however, it is difficult for individual laboratories to obtain isotopes for all analytes due to cost and availability. Therefore, alternative IS selection is important for practicality. Here, we quantified diphenhydramine and dextromethorphan concentrations in plasma from several collection sites in a fatal intoxication case, and assessed various IS performance based on structural similarities and retention time.

**Methods:**

A mid-teenager died from intoxication of personally imported dextromethorphan and Over-the-counter diphenhydramine. To quantify these drugs, we selected morphine-*d*_3_, dihydrocodeine, diphenhydramine-*d*_3_, mianserin-*d*_3_, and diazepam-*d*_5_ as alternative IS and evaluated. After selecting the most suitable IS, we quantified dextromethorphan and diphenhydramine concentrations in twelve plasma samples from the victim by liquid chromatography-tandem mass spectrometry.

**Results:**

Recovery rates were 80.7–105.5%, except for morphine-*d*_3_ (47.8%) and dihydrocodeine (64.8%). Matrix effects were 75.7–103.2%. The intra-day accuracies and precisions were 86.4–119.5% and 0.27–12.2%, respectively. The inter-day accuracies were 81.2–119.8%, and the precisions were 0.80–9.44%. The validation study showed that diphenhydramine-*d*_3_ was the most suitable IS. Finally, plasma concentrations of dextromethorphan and diphenhydramine were 3.74–10.3 µg/mL and 15.6–52.9 µg/mL, respectively.

**Conclusions:**

The concentrations of both drugs in plasma samples were estimated to cause death. When using an alternative IS, a validation study is needed to select the optimal IS.

## Introduction

Abuse of over-the-counter medicines (OTC) is one of the serious health problems around the world. These drugs are easily affordable and accessible compared with illicit drugs and medications that require prescriptions to obtain. Furthermore, since most people have the experience of using these drugs to care for themselves, there is less resistance and sense of danger to their abuse. The Japanese lifetime prevalence of illegal drug use, though, is low compared with Europe and the US in 2019–2020 [1]. OTC abuse on the other hand is a growing social problem in Japan. Shimane et al. reviewed the ratios of main drugs used by drug-related mental disorder patients from 2012 to 2020 in Japan. These results showed that OTC percentage was dramatically increased from 2.7% (2012) to 15.7% (2020). In the same period, marijuana increased from 1.8% to 5.3%, and methamphetamine from 28.9% to 36.0% [1]. In OTC overdose (OD) cases, cough suppressors and cold medicines are commonly ingested; these medicines frequently contain drugs like diphenhydramine (DPH), dextromethorphan (DEX), dihydrocodeine (DHC), and chlorpheniramine. Some concomitant drugs affect these drug concentrations in body fluids. For example, tricyclic antidepressants increase DPH blood concentrations via the extended half-life of DPH [2]. Ontiveras et al. reported a fatal case caused by dysrhythmia secondary to chlorpheniramine-induced sodium channel blockade with DEX toxicity [3]. Recently, Zimmerman et al. reported a suicidal OD case of DPH and melatonin, a sleep aid OTC [4].

DPH is a histamine H_1_ blocker used to treat allergic symptoms. Although the receptor is located throughout the body, adverse effects of sedatives are caused by the drug’s interaction with the receptor within the central nervous system [4]. It can also cause anticholinergic effects by blocking muscarinic receptors, which lead to tachycardia, dry mouth, and blurry vision, and so on. DPH also interacts with potassium ion channels within the heart which can induce prolonged QT intervals and flattened T waves; these result in fatal side effects such as torsade de pointes and other arrhythmias [5, 6]. The average fatal concentrations in biological fluids are reported as 19 µg/mL (range 7–31 µg/mL) [7].

DEX is a synthetic codeine analog and an enantiomer of levomethorphan [7]. Although levomethorphan is subject to regulation in Japan, DEX is not controlled and is used in numerous OTC cough medications. In recent years, DEX has been abused as a recreational drug for its euphoric, hallucinogenic, and dissociative effects [8]. It is said that both DEX and its metabolite, dextrorphan cause effects by stimulating the phencyclidine-1 receptor in the *N*-methyl-*d*-aspartate (NMDA) complex. Their effects are similar to those of ketamine and phencyclidine. Fatal DEX concentration in blood for teenagers and adults are reviewed as 1.0–18 µg/mL [7].

Since it is popular to purchase OTCs at drug stores and pharmacies, they are taking measures to limit the number of such medicine packages of which customers can purchase at a time in order to prevent OD in Japan. However, it is not easy to control excessive purchases since OTCs are not controlled substances. In addition, in recent years, there have been sporadic cases of obtaining OTCs via the Internet, including personal imports, making them even more accessible and easier to OD [9, 10].

The decision to declare cause of death from drug intoxication requires identification and quantification of the drugs in biological samples such as blood. In such cases, internal standards (IS) are generally used to correct for variations in experiment procedures, extraction efficiencies, and injection volumes into analytical instruments between samples. Stable isotopes of the target drugs containing deuterium (*d*), ^13^C, and ^15^N are preferable IS for their similarity of physiochemical properties, such as solubility and ionization, to the target analytes. However, they are relatively costly and are not always commercially available for all compounds; it is almost impossible for individual laboratories to assemble stable isotope compounds for all target substances. Furthermore, even authentic compounds that are not regulated are very difficult to obtain in Japan, especially compounds related to a controlled one, such as cannabinoids. The availability of authentic standards and their stable isotopes is nearly impossible due to excessive self-regulation by customs and distributors. When stable isotopic compounds cannot be used, in many cases, each laboratory has a compound that is commonly used as a general IS. Structurally similar compounds not naturally present in the biological specimens are also employed in some cases [11, 12]. In this study, we used several compounds that are similar in structure and retention time (RT), as internal standard candidates to quantify DPH and DEX and evaluated their performance as IS.

## Materials and methods

### Chemicals and reagents

**Fig. 1:**
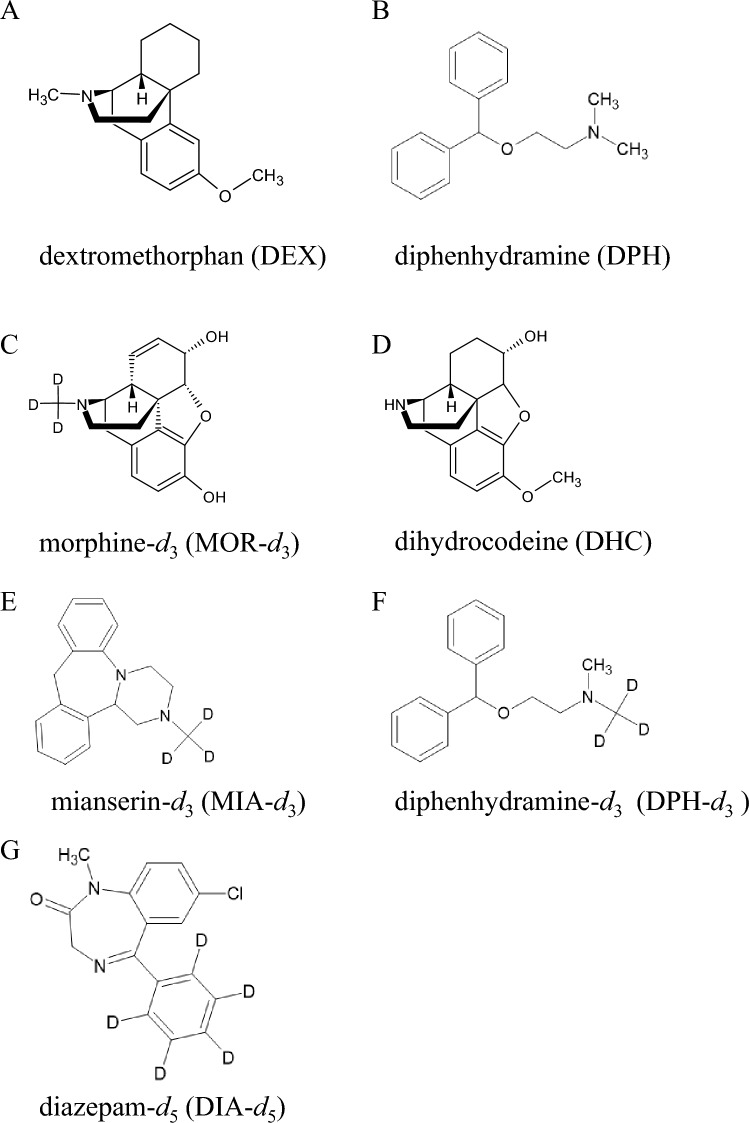
Structures of the target analytes and internal standard (IS) candidates. **A** dextromethorphan (DEX), **B** diphenhydramine (DPH), **C** morphine-*d*_3_ (MOR-*d*_3_), **D** dihydrocodeine (DHC), **E** mianserin-*d*_3_ (MIA-*d*_3_), **F** diphenhydramine-*d*_3_ (DPH-*d*_3_), and **G** diazepam-*d*_5_ (DIA-*d*_5_). **A**, **B** are target analytes; the others are IS candidates

Ultra-pure water (UW) and methanol for LC/MS, acetonitrile, and formic acid were purchased from Fujifilm Wako Pure Chemicals Ltd. (Osaka, Japan). DEX was obtained from Sigma-Aldrich Co. LLC (MO, USA). DPH was purchased from Wako Pure Chemicals Ltd. DPH-*d*_3_, morphine-*d*_3_ (MOR-*d*_3_), diazepam-*d*_5_ (DIA-*d*_5_), dihydrocodeine (DHC), and mianserin-*d*_3_ (MIA-*d*_3_) were from Cerilliant Corporation (TX, USA). The stable isotopic compounds and DHC were used as IS candidates. Ammonium formate was purchased from Sigma-Aldrich Co. LLC. The structures of analytes and IS candidates are shown in Fig. 1. DEX and DPH were dissolved with methanol to 100 µg/mL, and the others were prepared at ten µg/mL and stored at −30 ℃. Human plasma for blank specimens were obtained from Cosmo Bio Co., Ltd. (Tokyo, Japan). The blanks were confirmed not to contain any of the target analytes using LC/MS/MS.

### Sample preparation

For the experiments below, each plasma specimen from the deceased was diluted 2000-fold and 10,000-fold using commercially obtained plasma for quantifying DEX and DPH, respectively. Each authentic analyte and stable isotope was diluted by UW, and the final methanol content was under 0.1% in plasma specimens. Ten microliters of the IS mixture (final concentration of each IS, 5 ng/mL) and 10 µL of the DEX and DPH mix were added to 100 µL of diluted plasma specimens. Twenty microliters of saturated ammonium formate and 400 µL of acetonitrile were added to the 120 µL plasma specimens mentioned above. After vortexing for 60 s, the specimens were centrifuged at 15,000 × g for 5 min at 4 ℃. Then, after centrifugation, the specimens were separated into an organic layer and an aqueous layer. The organic layers were transferred into new tubes and dried under gentle nitrogen gas flow. The residues were reconstituted with 100 µL of the initial mobile phase for the liquid chromatograph tandem mass spectrometer (LC–MS-MS) and filtered through a Milex GV (PVDF, 0.22 µm) filter (Merk KGaA, Darmstadt, Germany). Five microliter aliquots were injected into the LC–MS-MS.

### Liquid chromatograph-tandem mass spectrometer (LC–MS-MS)

A Nexera X2 and LCMS-8050 were used as the LC–MS-MS system (Shimadzu Corp., Kyoto, Japan). Analysis was carried out by LabSolutions LCMS (Shimadzu). A Kinetex XB-C18 (2.6 µm, 150 mm × 2.1 mm) (Phenomenex Inc., CA, USA) was employed as the separation column. The flow rate was 0.3 mL/min. The mobile phase solutions were 0.1% formic acid and 10 mM ammonium formate in UW (A) and 0.1% formic acid and 10 mM ammonium formate in methanol (B). Gradient conditions were as follows: 5% of mobile phase B was linearly increased to 95% from 0 to 7.5 min, holding at 95% for 2.5 min. At 10 min, the mobile phase B concentration was decreased to 5% with a final hold of 5 min. The total run time was 15 min. The LC/MS/MS Method Package for Rapid Toxicology Screening (Shimadzu) was used for drug screening. Analytes were quantified using the multiple reaction monitoring (MRM) transitions represented in Table 1. Analyte concentrations were obtained using the area ratios of the analyte and IS or the area under the curves of analytes only.

**Table 1:** The retention times and multiple reaction monitoring transitions of the analytes

Compound Name	Retention time (min)	Precursor ion (*m/z*)	Product ion (*m/z*)	Q1 Pre Bias (V)	Collision energy (V)	Q3 Pre Bias (V)
			**215.0**	−17	−25	−15
DEX	5.40	272.1	171.0	−16	−39	−18
			212.95	−17	−27	−23
			**167.1**	−18	−17	−18
DPH	5.42	256.2	164.9	−19	−42	−11
			152.05	−10	−35	−30
			**201.15**	−18	−28	−21
MOR-*d*_3_	1.89	289.1	165.2	−17	−40	−17
			153.15	−18	−42	−29
			**198.9**	−17	−35	−24
DHC	2.77	302.1	227.1	−18	−27	−15
			171.0	−19	−44	−18
			**167.05**	−10	−15	−18
DPH-*d*_3_	5.42	259.1	152.0	−19	−38	−29
			165.0	−19	−44	−30
MIA-*d*_3_	5.43	268.15	**208.1**	−13	−21	−14
			61.1	−13	−27	−23
DIA-*d*_5_	6.81	290.0	**197.9**	−21	−33	−21
			227.15	−22	−29	−19

The product ions represented in bold characters were employed as quantifier ions and the others were qualifier ions

*DEX* dextromethorphan, *DPH* diphenhydramine, *MOR-d*_*3*_ morphine-*d*_3_, *DHC* dihydrocodeine, *DPH-d*_*3*_ diphenhydramine-*d*_3_, *MIA-d*_*3*_ mianserin-*d*_3_, *DIA-d*_*5*_ diazepam-*d*_5_

### Validation study

The limits of detection (LOD) and quantification (LOQ) were defined using signal-to-noise ratios of greater than 3 and 10, respectively, and were automatically evaluated by LabSolutions LCMS (Shimadzu). The calibration curves consisted of six concentration points (0.6, 1.5, 2, 3, 5, and 10 ng/mL in plasma, which correspond to 1.2, 3, 4, 6, 10, and 20 µg/mL for DEX and 6, 15, 20, 30, 50, and 100 µg/mL for DPH), each calibration point run in quintuplicate.

The recovery rates and matrix effects were examined at 1, 4.5, and 9 ng/mL for each target analyte as the quality control (QC) samples (These values correspond to 2, 9, 18 µg/mL for DEX and 10, 45, 90 µg/mL for DPH). The concentration of the various IS candidates was settled at 5 ng/mL. The recovery rates and matrix effects were calculated using the equations below.

$$Recovery\ rate \, \left(\%\right)=100\times \frac{Area\ under\ the\ curve\ of\ pre\ spike\ sample}{Area\ under\ the\ curve\ of\ post\ spike\ sample}$$

$$Matrix\ effect \, \left(\%\right)=100\times \frac{Area\ under\ the\ curve\ of\ post\ spike\ sample}{Area\ under\ the\ curve\ of\ authentic\ sample}$$

Intra-day and inter-day accuracies and precisions for QC samples of calibration curves were assayed at the same three concentration points above. Inter-day experiments were carried out over six successive days.

### Application

A high school student who is a friend of the victim called a karaoke box, and alerted the store staff that a boy who came in alone may try to commit suicide by ingesting drugs. A store staff searched throughout the store and found a mid-teenage male lying face up in one of the rental rooms with open eyes, not breathing, and without consciousness. Pills were scattered on the floor, and bottles labeled as DPH and DEX were found. DEX bottle was labeled in Chinese. An ambulance was called, and the store staff performed cardiac massage on the victim as instructed by the medical personnel. The victim was taken to the hospital in a state of cardiac arrest due to drugs overdose. Although he had been administrated saline, adrenaline, and sodium bicarbonate, he was confirmed dead after 2 h of he was found.

The autopsy was carried out at our university the following day. There was no trauma or lesions found to cause death; white solid substance was found in the stomach contents, and a light brown substance was adhered to the stomach wall. Blood samples were collected from 12 sampling sites during the autopsy. An alcohol test in whole blood from the right atrium was performed using gas chromatography, which revealed that alcohol concentration was under the detection limit. Drug screening by liquid chromatography-tandem mass spectrometry (LC/MS/MS) detected DEX and DPH in plasma. Sample collection and experiments were approved by the Institutional Review Board of Nagoya University Graduate School of Medicine (2015-0500).

## Results

We determined the LOD and LOQ of DEX and DPH in plasma. The LOD and LOQ were 0.2 ng/mL and 0.6 ng/mL for these drugs, respectively. Calibration curves with or without several internal standards (IS) were evaluated using correlation coefficients, and the results showed that they were all greater than 0.99 (Table 2).

**Table 2:** Calibration curve equations and correlation coefficients using different internal standards for the target analytes in plasma

Target analytes	IS	Calibration curves	Correlation coefficient (*R*^2^)
	None	y = 545237x-256530	0.991
	MOR-*d*_3_	y = 3.588x-1.813	0.991
DEX	DHC	y = 0.3860x-0.1733	0.992
	DPH-*d*_3_	y = 0.04354x-0.007403	0.995
	MIA-*d*_3_	y = 0.6493x-0.1669	0.992
	DIA-*d*_5_	y = 0.3516x-0.09423	0.993
	None	y = 1854592x-776892	0.995
	MOR-*d*_3_	y = 12.20x-5.527	0.995
DPH	DHC	y = 1.313x-0.5175	0.996
	DPH-*d*_3_	y = 0.1482x-0.01640	0.998
	MIA-*d*_3_	y = 2.209x-0.4400	0.996
	DIA-*d*_5_	y = 1.196x-0.2547	0.997

*DEX* dextromethorphan, *DPH* diphenhydramine, *MOR-d*_*3*_ morphine-*d*_3_, *DHC* dihydrocodeine, *DPH-d*_*3*_ diphenhydramine-*d*_3_, *MIA-d*_*3*_ mianserin-*d*_3_, *DIA-d*_*5*_ diazepam-*d*_5_

The recovery rates and matrix effects at 1, 4.5, and 9 ng/mL for the target drugs in plasma were evaluated as the QC. The recovery rates for DEX were 102.1% (1 ng/mL), 86.6% (4.5 ng/mL), and 82.6% (9 ng/mL). For DPH, the values were 99.2% (1 ng/mL), 90.0% (4.5 ng/mL), and 80.7% (9 ng/mL) (Table 3). The matrix effects of the target analytes were as follows: 103.2% (1 ng/mL), 96.5% (4.5 ng/mL), and 97.6% (9 ng/mL) for DEX, and 102.6% (1 ng/mL), 97.4% (4.5 ng/mL), and 96.8% (9 ng/mL) for DPH (Table 3). The recovery rates of IS at five ng/mL were 47.8% (MOR-*d*_3_), 64.8% (DHC), 95.3% (DPH-*d*_3_), 105.5% (MIA-*d*_3_), and 95.0% (DIA-*d*_5_) (Table 3). Matrix effects of the IS at the same concentrations were 96.1% (MOR-*d*_3_), 101.1% (DHC), 96.5% (DPH-*d*_3_), 75.7% (MIA-*d*_3_), and 83.5% (DIA-*d*_5_) (Table 3).

**Table 3:** The recovery rates and matrix effects of the target analytes and ISs

DEX	1 ng/mL	4.5 ng/mL	9 ng/mL
Recovery rate (%)	102.1	86.6	82.6
Matrix effect (%)	103.2	96.5	97.6

DPH	1 ng/mL	4.5 ng/mL	9 ng/mL
Recovery rate (%)	99.2	90.0	80.7
Matrix effect (%)	102.6	97.4	96.8

ISs at 5 ng/mL in plasma	MOR-*d*_3_	DHC	DPH-*d*_3_	MIA-*d*_3_	DIA-*d*_5_
Recovery rate (%)	47.8	64.8	95.3	105.5	95.0
Matrix effect (%)	96.1	101.1	96.5	75.7	83.5

*DEX* dextromethorphan, DPH diphenhydramine, *MOR-d*_*3*_ morphine-*d*_3_, *DHC* dihydrocodeine, *DPH-d*_*3*_ diphenhydramine-*d*_3_, *MIA-d*_*3*_ mianserin-*d*_3_, *DIA-d*_*5*_ diazepam-*d*_5_

Validation study revealed that intra-day accuracies and precisions for DEX in plasma were 86.4–119.5% and 0.70–12.2%, respectively, and 89.8–114.1% and 0.27–7.60%, respectively, for DPH (Table 4). The inter-day accuracies and precisions for the analytes were as follows: 81.2–119.8% for accuracies and 3.39–9.44% for precisions for DEX, 87.3–114.9% for accuracies and 0.80–6.43% for precisions for DPH (Table 5). Considering the results of the above validation studies, we selected DPH-*d*_3_ as the optimal IS for quantifying DEX and DPH in this study.

**Table 4:** Intra-day accuracies and precisions in plasma

DEX Intra-day accuracies (%)			
Target analyte/ISs	1 ng/mL	4.5 ng/mL	9 ng/mL
DEX/None	119.5	91.4	87.7
DEX/MOR-*d*_3_	119.1	89.9	86.4
DEX/DHC	115.7	93.0	89.0
DEX/DPH-*d*_3_	100.5	100.0	94.2
DEX/MIA-*d*_3_	103.5	93.1	86.4
DEX/DIA-*d*_5_	107.5	97.1	90.7

DEX Intra-day precisions (%)			
Target analyte/ISs	1 ng/mL	4.5 ng/mL	9 ng/mL
DEX/None	0.98	5.79	5.94
DEX/MOR-*d*_3_	1.18	5.99	6.99
DEX/DHC	0.70	5.85	5.60
DEX/DPH-*d*_3_	1.17	6.00	6.16
DEX/MIA-*d*_3_	4.06	12.2	9.60
DEX/DIA-*d*_5_	1.43	6.22	6.84

DPH Intra-day accuracies (%)			
Target analyte/ISs	1 ng/mL	4.5 ng/mL	9 ng/mL
DPH/None	114.1	94.0	91.2
DPH/MOR-*d*_3_	113.6	92.4	89.8
DPH/DHC	110.0	95.7	92.5
DPH/DPH-*d*_3_	94.2	103.1	98.0
DPH/MIA-*d*_3_	97.4	95.9	89.8
DPH/DIA-*d*_5_	101.6	100.1	94.3

DPH Intra-day precisions (%)			
Target analyte/ISs	1 ng/mL	4.5 ng/mL	9 ng/mL
DPH/None	0.27	0.49	0.47
DPH/MOR-*d*_3_	1.27	0.89	1.21
DPH/DHC	0.84	0.53	0.82
DPH/DPH-*d*_3_	0.74	0.40	0.54
DPH/MIA-*d*_3_	5.33	7.60	6.59
DPH/DIA-*d*_5_	2.38	0.42	2.57

*DEX* dextromethorphan, *DPH* diphenhydramine, *MOR-d*_3_ morphine-*d*_3_, *DHC* dihydrocodeine, *DPH-d*_*3*_ diphenhydramine-*d*_3_, *MIA-d*_3_ mianserin-*d*_3_, *DIA*-*d*_*5*_ diazepam-*d*_5_

**Table 5:** Inter-day accuracies and precisions in plasma

DEX Inter-day accuracies (%)			
Target analyte/ISs	1 ng/mL	4.5 ng/mL	9 ng/mL
DEX/None	119.8	89.3	83.5
DEX/MOR-*d*_3_	118.4	86.9	81.6
DEX/DHC	113.6	90.7	84.0
DEX/DPH-*d*_3_	102.5	97.3	90.0
DEX/MIA-*d*_3_	107.9	91.6	85.5
DEX/DIA-*d*_5_	103.0	87.6	81.2

DEX Inter-day precisions (%)			
Target analyte/ISs	1 ng/mL	4.5 ng/mL	9 ng/mL
DEX/None	3.49	4.98	3.49
DEX/MOR-*d*_3_	4.05	5.37	6.14
DEX/DHC	3.67	5.88	3.76
DEX/DPH-*d*_3_	5.22	5.54	3.39
DEX/MIA-*d*_3_	7.12	9.44	6.64
DEX/DIA-*d*_5_	4.60	7.92	7.64

DPH Inter-day accuracies (%)			
Target analyte/ISs	1 ng/mL	4.5 ng/mL	9 ng/mL
DPH/None	114.9	93.0	89.8
DPH/MOR-*d*_3_	113.4	90.4	87.8
DPH/DHC	108.4	94.4	90.3
DPH/DPH-*d*_3_	96.7	101.7	97.0
DPH/MIA-*d*_3_	102.3	95.5	92.1
DPH/DIA-*d*_5_	97.7	91.3	87.3

DPH Inter-day precisions (%)			
Target analyte/ISs	1 ng/mL	4.5 ng/mL	9 ng/mL
DPH/None	2.35	1.21	1.46
DPH/MOR-*d*_3_	3.82	3.96	5.12
DPH/DHC	3.73	3.04	1.80
DPH/DPH-*d*_3_	3.69	0.91	0.80
DPH/MIA-*d*_3_	6.03	6.43	5.92
DPH/DIA-*d*_5_	4.09	5.70	5.65

*DEX* dextromethorphan, DPH diphenhydramine, *MOR-d*_*3*_ morphine-*d*_3_, *DHC* dihydrocodeine, *DPH-d*_*3*_ diphenhydramine-*d*_3_, *MIA-d*_*3*_ mianserin-*d*_3_, *DIA-d*_*5*_ diazepam-*d*_5_

The quantified DEX and DPH concentrations in plasma samples collected from various sampling sites are presented in Table 6. The DEX concentrations were 9.11 µg/mL (Abdominal aorta), 8.06 µg/mL (Aorta), 8.92 µg/mL (Heart), 8.35 µg/mL (Inferior vena cava), 7.74 µg/mL (Left atrium), 6.79 µg/mL (Left pulmonary vein), 7.63 µg/mL (Right atrium), 10.3 µg/mL (Right femoral vein), 3.74 µg/mL (Right genu vein), 8.86 µg/mL (Subclavian vein), 6.52 µg/mL (Pulmonary artery), and 4.31 µg/mL (Right femoral artery) (Table 6). DPH concentrations were as follows: 41.7 µg/mL (Abdominal aorta), 38.6 µg/mL (Aorta), 47.7 µg/mL (Heart), 43.5 µg/mL (Inferior vena cava), 46.4 µg/mL (Left atrium), 50.6 µg/mL (Left pulmonary vein), 42.9 µg/mL (Right atrium), 52.9 µg/mL (Right femoral vein), 15.6 µg/mL (Right genu vein), 32.3 µg/mL (Subclavian vein), 36.5 µg/mL (Pulmonary artery), and 31.5 µg/mL (Right femoral artery) (Table 6).

**Table 6:** Quantified DEX and DPH concentrations in plasma from various sampling sites using DPH-*d3*

Sampling sites	DEX/DPH-*d*_3_ (µg/mL ± S.D.)	DPH/DPH-*d*_3_ (µg/mL ± S.D.)
Abdominal aorta	9.11 ± 0.75	41.7 ± 0.28
Aorta	8.06 ± 0.46	38.6 ± 0.20
Heart	8.92 ± 0.60	47.7 ± 0.26
Inferior vena cava	8.35 ± 0.60	43.5 ± 0.30
Left atrium	7.74 ± 0.58	46.4 ± 0.25
Left pulmonary vein	6.79 ± 0.60	50.6 ± 0.25
Right atrium	7.63 ± 0.54	42.9 ± 0.22
Right femoral vein	10.3 ± 0.84	52.9 ± 0.22
Right genu vein	3.74 ± 0.33	15.6 ± 0.13
Subclavian vein	8.86 ± 0.09	32.3 ± 0.40
Pulmonary artery	6.52 ± 0.41	36.5 ± 0.12
Right femoral artery	4.31 ± 0.34	31.5 ± 0.17

*DEX* dextromethorphan, *DPH* diphenhydramine, *DPH-d*_3_ diphenhydramine-*d*_3_, *S.D.* standard deviation

## Discussion

This study evaluated the performance of several IS candidates. The recovery rates of DPH and DEX were 80.7% to 102.1%. Those of DPH-*d*_3_, MIA-*d*_3_, and DIA-*d*_5_ at a concentration of 5 ng/mL were 95.0–105.5%. The recovery rates were comparable between these IS candidates and the target drugs. However, the recovery rates of MOR-*d*_3_ (47.8%) and DHC (64.8%) were lower compared to the values of DPH and DEX. Although the structures of MOR-*d*_3_ and DHC are similar to DEX, the retention times of these IS candidates were shorter than that of DEX due to their hydrophilic functional groups. Then, the log P of each compound was estimated by ALOGPS 2.1 as follows: 3.44 (DPH and DPH-*d*_3_), 3.75 (DEX), 0.99 (MOR-*d*_3_), 1.58 (DHC), 3.52 (MIA-*d*_3_), and 2.63 (DIA-*d*_5_) [13]. These values of MOR-*d*_3_ and DHC also represent higher hydrophilic properties of these compounds compared with the others.

It is possible that MOR-*d*_3_ and DHC were distributed more into the aqueous layer than other compounds during the extraction process, as we performed analyte extraction using acetonitrile and an aqueous ammonium formate solution and recovered the compounds in the organic layer. Matrix effects of all compounds, however, were 75.7–101.1%, including MOR-*d*_3_ and DHC.

The Guidelines for Quality Control in Forensic-Toxicological Analyses, published by the Society of Toxicological and Forensic Chemistry (GTFCh), recommend that variations in accuracy and precision values in the inter-day study are less than 15% or at least less than 20% [14]. According to the guideline, intra-day and inter-day variations of accuracies and precisions were acceptable for quantification at any QC concentration point, including those without an IS. However, using MOR-*d*_3_ and DHC as IS, as well as without any IS, the accuracy values at 1 ng/mL were high. As mentioned above, MOR-*d*_3_ and DHC had lower recovery rates due to their high hydrophilicity; this property may have affected the variations in accuracy and precision values. The LOQ value, 0.6 ng/mL, was evaluated to determine whether it met the criteria of ≦ 20% RSD and ± 20% bias at LOQ, as outlined in the guidelines [14]. The accuracies and precisions at LOQ were as follows: 112.0% and 5.39% (DEX/DHC), 105.6% and 1.57% (DEX/DPH-*d*_3_), 118.8% and 3.19% (DEX/DIA-*d*_5_), and 95.9% and 1.66% (DPH/DPH-*d*_3_) met the criteria, respectively. Following the guidelines, we selected DPH-*d*_3_ as the optimal IS, as the accuracy and precision in both intra-day and inter-day studies were considered the most reliable values.

We then quantified the concentrations of DEX and DPH in postmortem plasma in our overdose case using DPH-*d*_3_. Basalt reviewed three studies of acute DEX fatal cases, and the plasma DEX concentrations were 0.10–0.95 µg/mL (7 infants), 1.0–3.2 µg/mL (5 teenagers), and 3.3–9.2 µg/mL (2 adults) (7). In our study, DEX concentrations in plasma ranged from 3.74 to 10.3 µg/mL, indicating that DEX was present at potentially fatal concentrations in all plasma samples. Logan et al. reported on fatal overdose cases where DEX was obtained over the Internet [9]. The concentrations of DEX in postmortem blood were 950–3,230 ng/mL, and three of them contained DPH; two cases also detected cannabinoids, and one had trace alprazolam. The five case victims were all teenagers and obtained DEX from the same Internet supplier. In our case, the victim also seemed to have purchased and personally imported DEX via the Internet since the drug label was written in Chinese. Personal importation of therapeutic drugs is necessary for the continuation of treatment received abroad and for the use of drugs that are not yet approved in Japan. On the other hand, it also facilitates access to drugs for OD purposes, as in our present case. More strict regulations or licensing for suppliers may become necessary to prevent the acquisition of drugs for OD. Hasuwa et al. reported a single DEX overdose case and the concentrations of the drug in plasma and urine were 25 µg/mL and 111 µg/mL, respectively [8]. For most OD cases, multiple drugs are ingested, especially using OTC; the authors claimed that it was the first reported case of a single OD by DEX in Japan.

The quantified DPH concentrations in plasma in our study range from 15.6 to 52.9 µg/mL. Reported DPH concentrations in plasma in fatal cases are around 19 µg/mL (range 7 to 31 µg/mL) [7]. Oritani et al. reported the distribution of DPH in several biological fluids and solid tissues in an OD case by ingestion of motion sickness medications containing DPH [15]. DPH concentration in the right heart blood was 6.56 µg/mL [15]. The concentrations in the reproductive organs, such as the prostate and the right and left testes, were 73.42 µg/mL, 30.09 µg/mL, and 28.23 µg/mL, respectively, which were relatively higher than those in other solid tissues [15]. In their report, postmortem biochemical analysis revealed that C-reactive protein and neopterin, biomarkers of systemic inflammation, were elevated. Creatinine, myoglobin, and urea nitrogen levels in the blood were also elevated, indicating skeletal muscle damage and renal dysfunction. Therefore, the authors concluded the cause of death was rhabdomyolysis. Based on previous reports, our quantified DPH concentrations can cause death, just as DEX. DPH intoxications are typically caused by ingesting the drug, as in this study. Recently, Kusano et al. reported an unusual fatal case of an adult male using DPH as a topical medicine [16]. The DPH concentrations in plasma and urine were 0.44 µg/mL and 2,500 µg/mL, respectively. There were color differences between the right and left cardiac blood, and there were no observations of cardiovascular events that attributed to fatality; the authors reported that the cause of death was presumed to be hypothermia following a cascade of atypical events.

Cytochrome p450 (CYP) 2D6 metabolizes DEX, and the enzyme is known to have polymorphism [15, 17]. When the activity is low, the blood concentrations of the drug are excessively elevated, even at therapeutic doses [17, 18]. Furthermore, DPH is known to inhibit CYP2D6, and the combinational ingestion of DPH and DEX may cause an excessive increase in the concentration of DEX in the blood [17, 19]. Adverse effects, therefore, may occur even when dosage and administration are adhered to, depending on the concomitant drugs and genetic background of the individual. In our case, the concentrations of DEX must have been raised as the effect of CYP2D6 inhibition by DPH since the victim had ingested both drugs to commit suicide. These detrimental effects rationalize education for the public to adhere to dosage instructions as well as the symptoms of its side effects to avoid such severe adverse effects, including death, even for OTC drugs.

DEX and DPH are reported to exhibit postmortem redistribution. Han et al. reviewed three individual reports that the cardiac and femoral blood concentration DPH ratios were 1.1–2.4 (0.3–21) and exhibited their data as 2.09 (0.77–5.83) [20]. Basalt documented postmortem redistributions of DPH and DEX; the review showed that DPH’s heart/femoral vein blood ratios were 2.4 (0.4–6.0) and 2.3 (0.8–2.1) in 32 and 38 cases, respectively [7]. The DPH ratio of heart/subclavian blood concentrations was 1.1 (0.3–4.3) in 38 deaths. Other studies reported that DPH concentration in cardiac blood was higher than in peripheral blood [21, 22]. DEX ratios of heart/femoral vein blood concentrations were 2.0 (1.0–3.5) and 1.7 (1.0–3.2) in 5 and 11 cases, respectively [7]. Han et al. and Abdelaal et al. also reported a DEX ratio of 2.00 [20, 22].

Our results showed that the DPH ratio of heart to right femoral vein blood concentration was 0.9, and the ratio of heart to subclavian vein blood concentration was 1.5. The DEX concentration ratio of heart to right femoral vein blood in our study was around 1.0; our results were considered to be within the range of variability reported by previous studies.

## Conclusions

The quantified plasma DEX and DPH concentrations in our case were enough to cause death. Our results suggest that DPH-*d*_3_ is the most suitable for IS in this study. Structural similarity is not necessarily required when the hydrophilic or hydrophobic properties of the IS differ; therefore, validation studies are preferred for selecting the optimal alternative IS for individual target analytes.

## References

[1] Shimane T, Inoura S, Matsumoto T (2021) Proposed indicators for Sustainable Development Goals (SDGs) in drug abuse fields based on national data from Japan. J Natl Inst Public Health 70:252–261

[2] Yokoyama K, Kaizaki-Mitsumoto A, Numazawa S, Mamada M (2024) Diphenhydramine intoxication with blood extended half-life and a false positive result for tricyclic antidepressants. Cureus 16(6):e63540. 10.7759/cureus.63540 39086780 10.7759/cureus.63540 PMC11289077

[3] Ontiveros S, Cantrell L (2021) Fatal cold medication poisoning in an adolescent. Am J Emerg Med 2022(52):269.e1-269.e2. 10.1016/j.ajem.2021.08.043 10.1016/j.ajem.2021.08.043 34454805

[4] Zimmerman JT, Schreiber SJ, Huddle LN (2023) Case report of lethal concentrations of the over-the-counter sleep aids diphenhydramine and melatonin. Am J Forensic Med Pathol 44(3):227–230. 10.1097/PAF.0000000000000833 37195072 10.1097/PAF.0000000000000833 PMC10430669

[5] Radovanovic D, Meier PJ, Guirguis M, Lorent JP, Kupferschmidt H (2000) Dose-dependent toxicity of diphenhydramine overdose. Hum Exp Toxicol 19(9):489–495. 10.1191/096032700671040438 11204550 10.1191/096032700671040438

[6] Taglialatela M, Timmerman H, Annunziato L (2000) Cardiotoxic potential and CNS effects of first-generation antihistamines. Trends Pharmacol Sci 21(2):52–56 10664607 10.1016/s0165-6147(99)01437-6

[7] Basalt R, ed. (2020) Disposition of toxic drugs and chemicals in man. 12th ed. Foster City, CA: Biomedical Publications; pp. 604–606 (dextromethorphan), pp. 682–684 (diphenhydramine).

[8] Hasuwa K, Inoue N, Tamura S, Terazawa I, Yuui K, Kudo R, Kasuda S (2024) An autopsy case of a young man with a single overdose of dextromethorphan. Leg Med (Tokyo) 70:102470. 10.1016/j.legalmed.2024.102470 38878748 10.1016/j.legalmed.2024.102470

[9] Logan BK, Goldfogel G, Hamilton R, Kuhlman J (2009) Five deaths resulting from abuse of dextromethorphan sold over the internet. J Anal Toxicol 33(2):99–103. 10.1093/jat/33.2.99 19239735 10.1093/jat/33.2.99

[10] Karakis I, Kostandini G, Tsamakis K, Zahirovic-Herbert V (2024) The association of broadband internet use with drug overdose mortality rates in the united states: cross-sectional analysis. Online J Public Health Inform 26(16):e52686. 10.2196/52686 10.2196/52686 PMC11237777 38922664

[11] Natori Y, Kamioka S, Yoshimoto T, Ishii A (2022) A simple and rapid method for quantifying aconitines and their metabolites in whole blood by modified QuEChERS and liquid chromatography/tandem mass spectrometry (LC/MS/MS). Forensic Sci Int 341:111475. 10.1016/j.forsciint.2022.111475 36202020 10.1016/j.forsciint.2022.111475

[12] Kaneko R, Hattori S, Furuta S, Hamajima M, Hirata Y, Watanabe K, Seno H, Ishii A (2006) Sensitive analysis of aconitine, hypaconitine, mesaconitine and jesaconitine in human body fluids and Aconitum tubers by LC/ESI-TOF-MS. J Mass Spectrom 41(6):810–814. 10.1002/jms.1038 16770829 10.1002/jms.1038

[13] Virtual Computational Chemistry Laboratory [online]; https://vcclab.org/lab/alogps/. Accessed 16 June 2025

[14] Paul LD, Musshoff F, Aebi B, Auwaeter V, Peters KT (2018) GTFCh guideline for quality control in forensic toxicological analyses. Toxichem Krimtech 85:2–6

[15] Oritani S, Michiue T, Chen JH, Tani N, Ishikawa T (2017) Biodistribution of diphenhydramine in reproductive organs in an overdose case. Hum Cell 30(2):106–116. 10.1007/s13577-016-0151-9 27838883 10.1007/s13577-016-0151-9

[16] Kusano M, Fujishiro M, Hashimoto M, Ng MJ, Yoshida R, Narita SI, Nakauchi A, Sato K, Tachi Y, Matsuyama T (2023) An unusual case of fatal hypothermia involving topical diphenhydramine. Forensic Toxicol 41(1):158–163. 10.1007/s11419-022-00637-7 36652061 10.1007/s11419-022-00637-7

[17] Grzegorzewski J, Brandhorst J, König M (2022) Physiologically based pharmacokinetic (PBPK) modeling of the role of CYP2D6 polymorphism for metabolic phenotyping with dextromethorphan. Front Pharmacol 24(13):1029073. 10.3389/fphar.2022.1029073 10.3389/fphar.2022.1029073 PMC9637881 36353484

[18] Marasanapalle VP, Masimirembwa C, Sivasubramanian R, Sayyed S, Weinzierl-Hinum A, Mehta D, Kapungu NN, Kanji C, Thelingwani R, Zack J (2024) Investigation of the differences in the pharmacokinetics of CYP2D6 substrates, desipramine, and dextromethorphan in healthy African subjects carrying the allelic variants CYP2D6*17 and CYP2D6*29, when compared with normal metabolizers. J Clin Pharmacol 64(5):578–589. 10.1002/jcph.2366 37803948 10.1002/jcph.2366

[19] Akutsu T, Kobayashi K, Sakurada K, Ikegaya H, Furihata T, Chiba K (2007) Identification of human cytochrome P450 isozymes involved in diphenhydramine N-demethylation. Drug Metab Dispos 35(1):72–78. 10.1124/dmd.106.012088 17020955 10.1124/dmd.106.012088

[20] Han E, Kim E, Hong H, Jeong S, Kim J, In S, Chung H, Lee S (2012) Evaluation of postmortem redistribution phenomena for commonly encountered drugs. Forensic Sci Int 219(1–3):265–271. 10.1016/j.forsciint.2012.01.016 22284073 10.1016/j.forsciint.2012.01.016

[21] Tominaga M, Michiue T, Oritani S, Ishikawa T, Maeda H (2016) Evaluation of postmortem drug concentrations in bile compared with blood and urine in forensic autopsy cases. J Anal Toxicol 40(5):367–373. 10.1093/jat/bkw028 27185819 10.1093/jat/bkw028

[22] Abdelaal GMM, Hegazy NI, Etewa RL, Elmesallamy GEA (2023) Postmortem redistribution of drugs: a literature review. Forensic Sci Med Pathol. 10.1007/s12024-023-00709-z 37715933 10.1007/s12024-023-00709-z PMC11790690

